# Cysteine 95 and other residues influence the regulatory effects of Histidine 69 mutations on Human Immunodeficiency Virus Type 1 protease autoprocessing

**DOI:** 10.1186/1742-4690-7-24

**Published:** 2010-03-23

**Authors:** Liangqun Huang, Alyssa Hall, Chaoping Chen

**Affiliations:** 1 Department of Biochemistry and Molecular Biology, Colorado State University, Fort Collins, Colorado, USA

## Abstract

**Background:**

Regulated autoprocessing of HIV Gag-Pol precursor is required for the production of mature and fully active protease. We previously reported that H69E mutation in a pseudo wild type protease sequence significantly (>20-fold) impedes protease maturation in an *in vitro *autoprocessing assay and in transfected mammalian cells.

**Results:**

Interestingly, H69E mutation in the context of a laboratory adapted NL4-3 protease showed only moderate inhibition (~4-fold) on protease maturation. There are six point mutations (Q7K, L33I, N37S, L63I, C67A, and C95A) between the NL4-3 and the pseudo wild type proteases suggesting that the H69E effect is influenced by other residues. Mutagenesis analyses identified C95 as the primary determinant that dampened the inhibitory effect of H69E. L63 and C67 also demonstrated rescue effect to a less extent. However, the rescue was completely abolished when H69 was replaced by aspartic acid in the NL4-3 backbone. Charge substitutions of surface residues (E21, D30, E34, E35, and F99) to neutral or positively charged amino acids failed to restore protease autoprocessing in the context of H69E mutation.

**Conclusions:**

Taken together, we suggest that residue 69 along with other amino acids such as C95 plus L63 and C67 to a less extent modulate precursor structures for the regulation of protease autoprocessing in the infected cell.

## Background

**Figure 1:**
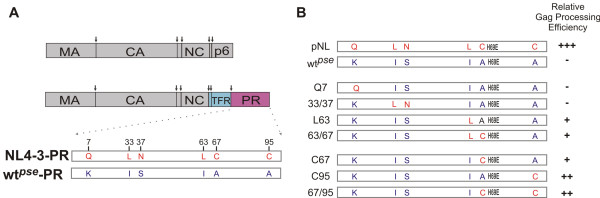
**Schematic illustration of constructs with or without H69E mutation**. **(A) **Organization of structural domains in the Gag and Gag-PR polyproteins: MA, matrix; CA, capsid (p24); NC, nucleocapsid; p6, late domain protein; TFR, transframe region; PR, protease. Straight arrows indicate the protease cleavage sites. Amino acids that are different between NL4-3 and wt^*pse *^proteases are denoted. **(B) **Schematic summary on H69E containing mutants and their relative Gag processing efficiencies.

Human immunodeficiency virus 1 (HIV-1) is a member of the lentivirus genus in the retroviradae superfamily. In the HIV infected cell, the unspliced genomic RNA also serves as mRNA for translation of two polyproteins: Gag and Gag-Pol [1,2]. Gag polyprotein is the primary viral determinant responsible for the assembly and release of progeny virions [3,4]. Gag-Pol polyprotein is produced as a result of regulated frameshifting that reads through the stop codon in the Gag reading frame [5,6]. In the Gag-Pol precursor, HIV protease is flanked N-terminally by the transframe region (TFR) (Figure 1A) and C-terminally by the reverse transcriptase [5,7]. The embedded precursor protease has an intrinsic ability to catalyze cleavages of a few sites in Gag and Gag-Pol polyproteins [8-10], but the full proteolytic activity is only associated with the mature protease after it is liberated from the precursor as a result of autoprocessing. The N-terminal cleavage is critical for protease maturation [5,11] since blocking the N-terminal cleavage abolishes the production of mature protease [10,12]. In contrast, mutations blocking the C-terminal cleavage have no significant influence on protease activity [13,14].

The mature protease recognizes and cleaves at least 10 different sites in Gag and Gag-Pol polyproteins [15,16]. These sites are processed at rates that vary up to 400-fold *in vitro *[17,18], probably due to the diversity of target sequences [19]. Among the five canonical HIV-1 Gag processing sites, the p2/NC site appears to be the preferred substrate as both protease precursor and mature protease can cleave this site with high efficiency [9,20]. In contrast, mature protease is required for the cleavage at the CA/p2 site [17,21]. Accurate and precise protease processing is absolutely required for the production of infectious progeny virions. Mutations that alter the time of processing or the order in which these sites are cleaved, or that produce incorrect cleavage at individual sites, cause the release of aberrant virions that are significantly less infectious [22-25].

The mature HIV protease is composed of 99 amino acids and is a member of the aspartyl protease family [7,26,27]. Unlike the cellular aspartic proteases that are active monomers, mature HIV protease exists as stable dimers (*K*_d _< 5 nM) with the catalytic site formed at the dimer interface by two aspartic acids; each is contributed by one monomer [5]. Mutations that alter the aspartic acid to either asparagine or alanine abolish protease activity *in vitro *and *in vivo *[27-30]. In contrast to mature proteases that are stable dimers, protease precursors containing the N-terminal TFR have a much higher dimer dissociation constant (*K*_d _> 500 μM) and exhibit very low catalytic activity [5,11]. Transient protease precursor dimerization coupled with the N-terminal cleavage is concomitant with the formation of stable dimers and the appearance of full catalytic activity when purified protease precursors are refolded *in vitro *[31,32] - a process defined as autocatalytic maturation or autoprocessing [5].

A pseudo wild type protease, which bears six point mutations (Q7K, L33I, N37S, L63I, C67A, and C95A) compared to the NL4-3 protease, has been previously optimized for NMR and kinetic studies of protease maturation [11]. Mutations Q7K, L33I, L63I minimize autoproteolysis; C67A and C95A prevent cysteine-thiol oxidation. We previously described that alteration of His 69, a surface residue of the mature protease, to glutamic acid in the pseudo wild type protease sequence significantly blocks precursor autoprocessing both in *E. coli *and in transfected mammalian cells [33]. Biochemical analyses indicate that the mature H69E protease displayed a slightly lower catalytic activity comparable to the wild type protease. However, *in vitro *autoprocessing of H69E precursor is drastically delayed, suggesting that H69E mutation may interfere with productive folding of the precursor. Interestingly, H69E mutation in the context of NL4-3 derived protease only demonstrated a moderate inhibitory effect on protease maturation. We sought here to define residues that contribute to the differential impacts on precursor autoprocessing. This information would provide insights into the molecular mechanism that regulates protease autoprocessing.

## Results

### H69E mutation displayed different effects under two different contexts

In our previous report, H69E and other mutations were constructed in the context of a pseudo wild type (wt^*pse*^) protease sequence, in which H69E significantly impedes precursor autoprocessing. Compared to the laboratory adapted NL4-3 derived protease, the pseudo wild type protease contains six point mutations (Figure 1A), but otherwise displays enzymatic kinetics similar to the wild type protease [34]. Mutations Q7K, L33I, and L63I are known to minimize autoproteolysis; and C67A/C95A mutations prevent aggregation of *E. coli *expressed protease mediated by cysteine thiol oxidation. To further understand the inhibition mechanism of H69E on protease autoprocessing, we first sought to examine the effects of H69E in the context of NL4-3 protease.

**Figure 2:**
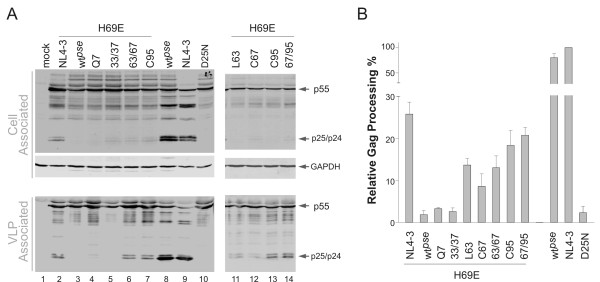
**Cysteine 95 and other residues dampened the inhibitory effect of H69E on protease autoprocessing in transfected mammalian cells**. **(A) **The indicated proviral DNAs were transfected into HEK 293T cells grown on 6-well plates with calcium phosphate. The total cell lysates and VLPs were prepared as described (Material and Methods) and subjected to western blot analysis. Mouse monoclonal anti-p24 antibody was used to detect proteins such as the full length Gag polyprotein (p55), CA-p2 intermediate (p25), and final processing product (p24) in the transfected cells and the released VLPs. The cell lysates blot was stripped and reprobed for GAPDH as loading controls. **(B) **Relative Gag processing efficiencies were quantified from three independent experiments and the bars represent standard deviations.

The previously described pNL-PR proviral construct was used to engineer the indicated mutations (Figure 1B), and the resulting plasmids were transfected into HEK 293T cells for the study. Approximately equal amounts of total Gag proteins were detected in cell lysates suggesting similar expression efficiencies mediated by the pNL-PR proviruses. Also, the amounts of virus-like particle (VLP) released into the culture medium were similar to each other, indicating these mutations have minimal impact on virion production. In the absence of any protease activity, as with D25N mutant, the full length Gag polyprotein (p55) is the predominant product in transfected cells and the released VLPs (Figure 2 lane 10). In the presence of mature proteases as a result of effective autoprocessing, p24 was detected as the predominant band with little p25 and p55 (Figure 2 lanes 8 and 9). Consistent with our previous report, VLPs produced by wt^*pse *^H69E contained predominantly the full length Gag polyprotein and no processed p24, indicating lack of mature protease activity (Figure 2, lane 3). Interestingly, VLPs as well as cell lysates made by NL4-3 H69E showed some p24 proteins, suggesting an association of mature protease activity in both. We quantified the ratio of p24 to total p24-containing proteins as a measure of relative Gag processing efficiency to indirectly reflect autoprocessing activity, and our data demonstrated that wt^*pse *^H69E mutation had <5% of the wild type processing activity, *i.e*. > 20-fold inhibition; while NL4-3 H69E showed ~25% of the wild type processing efficiency, *i.e*. ~4-fold inhibition (Figure 2B). Given that there are six point mutations between NL4-3 and wt^*pse *^protease, our data suggested that the inhibitory effect of H69E on protease autoprocessing is influenced by other residues.

### C95 and other residues dampened the inhibitory effect of H69E on protease autoprocessing

In order to define residues that rescued protease autoprocessing in the NL4-3 H69E construct, we engineered a panel of H69E proviruses replacing the six point mutations in the wt^*pse *^backbone with the corresponding NL4-3 amino acids individually or in combination (Figure 1B) and tested their Gag processing efficiencies to evaluate autoprocessing activities (Figure 2). The wt^*pse *^H69E mutants carrying NL4-3 Q7, L33/N37 demonstrated a phenotype very similar to the wt^*pse *^H69E, suggesting that these residues contributed minimally to the rescue effect. In contrast, wt^*pse *^H69E/A95C mutant, which contains single amino acid reversion at residue 95, showed a relative Gag processing activity close to NL4-3 H69E mutant, indicating that C95 could facilitate autoprocessing. Interestingly, the double mutation I63L/A67C also demonstrated rescued Gag processing to a less extent (Figure 2 lane 6). To further pinpoint the contributing residue(s), we mutated each residue individually, and the resulting constructs showed that both rescued the activity similarly to the double mutation (Figure 2A). Based on these observations, we suggested that cysteine 95 is the primary residue facilitating protease autoprocessing and the subsequent Gag processing; L63 and C67 can also rescue the H69E inhibitory effect to a less extent probably because of the fact that they are in the close proximity to H69 residue in primary sequence. The double mutations, L63/C67 and C67/C95, only showed a slight enhancement on protease activity compared to the single mutations, indicating a lack of synergistic effect. We interpreted that these residues are capable of facilitating autoprocessing independently to a certain extent and these enhancements might be parallel to each other and not additive.

### H69D mutation abolishes protease autoprocessing even in the context of NL4-3 PR backbone

**Figure 3:**
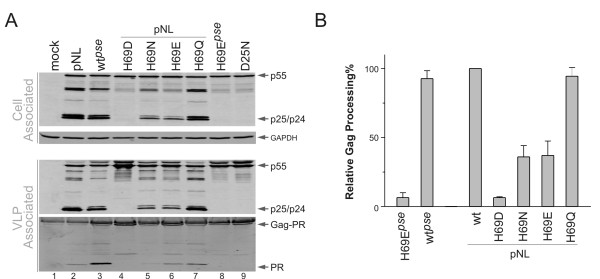
**Different substitutions of H69 have differential effects on protease maturation**. **(A) **HEK 293T cells grown on 6-well plates were transfected with the indicated proviral DNAs by calcium phosphate. The total cell lysates and VLPs were prepared as described (Material and Methods) and subjected to western blot analysis. Mouse monoclonal anti-p24 antibody was used to detect p24-containing proteins (p55, p25, and p24) in the transfected cells and the released VLPs. The cell lysates blot was stripped and reprobed for GAPDH as loading controls. VLP associated proteases were probed with polyclonal rabbit anti-PR antibodies. **(B) **Relative Gag processing efficiencies were quantified from three independent experiments and the bars represent standard deviations.

In addition to H69E mutation, a previous study using bacterially expressed Gag-Pol precursor demonstrated inhibition of protease autoprocessing by H69D; whereas changes to R, L, Y, N, and Q, individually, did not impair protease autoprocessing [29]. To compare H69E with H69D for their effects on protease maturation under the same context, we engineered a panel of mutations changing the parental H69 to D, N and Q individually in the pNL-PR backbone. As shown in Figure 3, VLPs produced by H69Q mutant displayed a p24 pattern similar to the wild-type control; and both H69N and H69E showed partial Gag processing activities. In contrast, H69D VLPs only contained the full length p55 precursor; no processed intermediates or p24 were detected (Figure 3 lane 4), which resembled the D25N negative control. This data further verified that aspartic acid at position 69 significantly blocks protease maturation even in the presence of L63, C67, and C95. It is interesting that H69D mutation displays a more drastic inhibitory effect than H69E considering the carboxyl side chain of aspartic acid is only shorter by one methyl group (-CH_2_) than that of glutamic acid. Quantitative analysis demonstrated relative Gag processing efficiencies following an order of wt ≅ H69Q > H69N, H69E >> H69D in VLPs produced from transfected mammalian cells (Figure 3B). By examining structures of these amino acids, it seemed that a combination of the carbonyl group and its close distance to the Cα plays a role in inhibiting protease maturation.

Steady state levels of mature protease detected in VLPs (Figure 3A, the bottom panel) also qualitatively correlated with the relative Gag processing activities (Figure 3B). A rabbit polyclonal anti-PR antibody detects both mature and precursor proteases, but the precursor band overlaps with a non-specific background band (Figure 3A lane 1), so we mainly focused on detection of mature protease. In VLPs produced by the wild type NL4-3 and wt^*pse*^, mature protease is the primary product, consistent with the high Gag processing efficiencies. The wt^*pse *^mature protease appeared to be more than the NL4-3 mature protease probably due to its higher stability because of the mutations engineered to reduce autoproteolysis. In VLPs produced from H69Q, mature protease was the primary form similar to the wild type control. Consistent with the partial Gag processing activities, H69E and H69N VLPs contained reduced amounts of mature protease as well as partially processed intermediates. In D25N, H69D, and wt^*pse *^H69E VLPs, minimal or no mature protease was detected; and the full length Gag-PR precursor appeared to be the predominant product.

### Charge substitutions of several residues did not rescue inhibition of H69E on protease maturation

**Figure 4:**
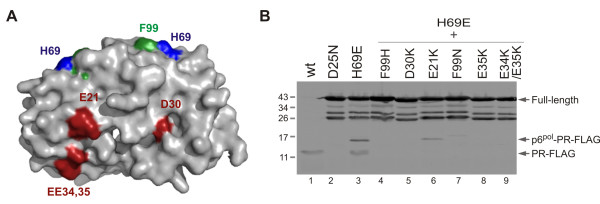
**Charge substitutions of surface residues did not restore the inhibitory effect of H69E on protease autoprocessing**. **(A) **Schematic presentation of the mature protease dimer (PDB 2PK6) with the surface residues that were tested in this report highlighted in red or green and histindine 69 in blue. **(B) **The pGEX-3X derived plasmids encoding for GST-TFR-PR-FLAG fusions bearing the indicated mutations were introduced into *E. coli *BL21(DE3) and induced for protein expression. The total lysates were prepared as described (Materials and Methods) and subjected to western blot analysis. A mouse anti-FLAG antibody was used to detect the full length precursor fusion, intermediates and mature protease (PR-FLAG). The denoted protein markers are in kDa for reference.

Our mutagenesis analyses demonstrated that the negatively charged carbonyl group at close proximity to the C_α _of residue 69 inhibits protease maturation. Our previous study also suggested that H69E mutation inhibits *in vitro *autoprocessing probably by affecting proper precursor folding. One speculation is that positively charged side chains of the parental residues (H69 or K69) interact with another negatively charged residue to facilitate proper folding; and the carbonyl group of H69E disrupts the electrostatic interaction. To test this possibility, we performed a small scale screening for potential H69 interacting residues using a previously reported precursor autoprocessing assay [33]. When expressed in *E. coli*, GST-TFR-PR-FLAG fusion precursor autoprocesses releasing mature protease that can be detected in total lysates by Western blot (Figure 4 lane 1). H69E mutation significantly inhibits protease maturation (lane 3). We chose to mutate five surface residues (four acidic acids plus F99 that is in close proximity to H69) individually in the H69E context to examine whether a neutral or positively charged residue at these positions could rescue protease autoprocessing by complementing mutations. Out of a total of 12 constructs (E21K, E21Q, D31K, D31N, E34K, E35K, E34K/E35K, F99K, F99N, F99Q, F99H, F99A), none of them reversed the inhibitory effect of H69E on protease maturation (not all the mutants are shown here) and many of them further suppressed autoprocess activity (Figure 4, lanes 4-9). Consequently, our limited screening was unable to define residues that might interact with H69, and further examinations would be necessary to identify how H69 regulates protease maturation.

## Discussion and Conclusions

Protease autoprocessing involves precursor dimerization and the N-terminal cleavage that releases mature protease. In the infected cell, this process is also temporally correlated with the virion egress event. However, the molecular and cellular mechanisms underlying this highly regulated process are poorly understood. We previously reported that H69E mutation in a pseudo wild type protease sequence abolishes protease autoprocessing in *E. coli *and in transfected mammalian cells [33]. The current study demonstrates that L63, C67, and C95 dampen the H69E inhibitory effect. The Levine group also suggested a possible inter-play between H69 and C67 using a model peptide spanning residues 59 to 75 more than a decade ago [35]. It is interesting to note that highly conserved HIV-1 protease cysteines are not required for the catalytic activity, nor contributed to the formation of intramolecular disulfide bonds. Instead, they are thought to participate in redox regulation of protease activity [36,37] via a yet-to-be-defined mechanism. Both C67 and C95 appear to be sensitive to oxidation with C95 seems more accessible than C67 [36,38]. Glutathionylation of C67 increases and stabilizes protease activity *in vitro*, whereas C95 glutathionylation abolishes protease activity [38]. Using immature HIV virions produced in the presence of protease inhibitors as a model system, Davis *et al*. demonstrated that immature virions made from a mutant lacking the two cysteines undergo protease maturation at a higher rate than the wild type immature virions following the removal of inhibitors [37]. Reducing agent DTT enhances protease maturation, and oxidizing agents delay protease maturation of the immature virions. These results suggest an oxidation-and-reduction cycle that is involved in regulation of protease autoprocess. We envision that oxidation of cysteines prevents protease precursor from pre-maturation by locking it in an inactive status in the infected cell. Upon virion release, other factors trigger the reduction reaction that restores free cysteines rendering protease activity. This cysteine modification cycle seems unnecessary for protease autoprocessing and mature protease activity as the pseudo wild type protease containing mutations C67A/C95A is able to process Gag polyprotein at levels comparable, yet slightly lower, to NL4-3 protease (Figure 2 and 3). However, in the context of H69E pseudo wild type protease, cysteine containing protease demonstrated a relative Gag processing activity higher than that lacking cysteines (Figure 2A). Therefore, the modification cycle might play an auxiliary role in concert with other regulation mechanisms to modulate protease autoprocessing.

Amino acid sequence alignment of HIV-1 proteases (HIV database - http://www.hiv.lanl.gov) indicates that residue 69 is mostly histidine or lysine and occasionally glutamine or tyrosine, which are neutral or positively charged. Previous studies [29,33] and current report also support the notion that a carbonyl group at close proximity to the Cα position of this residue inhibits protease autoprocessing. The H69 residue is exposed on the surface of mature protease dimer and is close to the C-terminus. It is intriguing that charge properties of a surface residue would have drastic effects on protease autoprocessing. Previous biochemical analyses demonstrated that H69E mutation significantly delays the TFR-PR precursor from autoprocessing in vitro; whereas the appropriately folded H69E mature protease only showed a slightly decreased catalytic activity [33]. This has led us to speculate that residue 69 is involved in autoprocessing by influencing precursor structure. We hypothesize that protease precursor undergoes conformational changes during autoprocessing and a carbonyl group close to the Cα of position 69 interferes with this pathway. It would be critical to identify residues that transiently interact with H69 during this process. Unfortunately, our limited screening was unable to define any of them. Extensive structural and biochemical analyses on the wild type and H69D precursor would be essential to provide insights into protease autoprocessing mechanisms.

## Methods

### DNA mutagenesis

Plasmids that were used in this report were generated with the standard molecular cloning procedures and the detailed sequence information is available upon request. Construction of pNL-PR was described previously [33], and all the pNL-PR mutants were derived from this vector by site-directed mutagenesis. Multiple D21, D30, E34, E35 and F99 substitutions were introduced into a pGEX-3X derived plasmid expressing GST-p6^pol^-PR^*pse*^-FLAG H69E was generated in a previous report [33]. All the plasmids were purified with QIAEX plasmid kits and verified by DNA sequencing.

### Cell culture, transfection and western blotting

Human embryonic kidney derived 293T cells (ATCC, Manassas, VA) were maintained in DMEM with 10% fetal bovine serum and transfected by calcium phosphate as previously described [33]. In brief, 293T cells were plated in 6-well plates the night before to give 50-60% confluence at the time of transfection. One hour prior to the transfection, chloroquine was added to each well to a final concentration of 25 uM. A total of 1 μg DNA in 131.4 μL of ddH_2_O was mixed with 18.6 μl 2 M CaCl_2 _to give a final volume of 150 μl. Then, 150 μl of 2 × HBS was added dropwise to the DNA solution while mixing by vortex. The resulting mixture was directly added to the culture cells. After 7-11 h of incubation, the culture medium was replaced with chloroquine-free DMEM.

Total cell lysates were prepared as described previously [33,39,40] to examine proteins in transfected cells. To examine proteins associated with the released virions, culture media collected from 11 h to 48 h post transfection was clarified of cell debris by a brief centrifugation (20,800 × g for 2 min at ambient temperature) and the supernatant was transferred to another tube and centrifuged at 20,800 × g for 3 h at 4°C to pellet virions. Virion pellets were resuspended in 40 μl of PBS for further analysis. About 1/6 of cell lysate made from each well was resolved through 10% SDS-PAGE and the proteins were transferred to a PVDF (Polyvinylidene Fluoride) membrane followed by western blot. Approximately one half of virus-like particles (VLPs) collected from each well were analyzed for p24 contents, and all the VLPs made from one well of a 6-well plate were used for protease detection. Mouse anti-HIV p24 antibodies (Cat# 3537) and rabbit anti HIV-1 protease serum (Cat# 4105) were obtained from the NIH AIDS research and reference program. Mouse anti-GAPDH (clone 6C5) antibodies (Fisher Scientific, Pittsburgh, PA) were used to reflect cell numbers. IR800 labelled goat anti mouse or rabbit secondary antibodies were purchased from Rockland Immunochemicals Inc (Gilbertsville, PA) for western detection with an Odyssey infrared dual laser scanning unit.

### Quantification of relative Gag processing activity

Western blot images that were captured by an Odyssey infrared dual laser scanning unit in tiff format were analyzed by Totallab software (Nonlinear Dynamics Inc., Newcastle upon Tyne, UK). Total pixel volume (less than the saturation threshold) of each band was quantified to represent band intensity that is assumed to be proportional to protein amounts as the blot was detected by monoclonal antibodies. The anti-p24 antibody is able to detect the full length (p55) Gag polyprotein as well as p25 (CA-p2), a processing intermediate, and p24, the final cleavage protein. Because the production of p24 from p25 is solely dependent on mature protease, the amounts of p24 in VLPs quantitatively correlate with the amounts of mature protease that indirectly reflect precursor maturation efficiencies. In this report, we calculated the ratio of p24/(p24+p25+p55) as a measure of Gag processing efficiency to indirectly represent autoprocessing activities with the value obtained from the wild type pNL-PR VLPs set as 100% for normalization.

### Protease autoprocessing in E. coli

The pGEX-3X derived plasmids were transformed into BL21 cells (Novagen, San Diego, CA) and the individual colony was grown in LB medium at 37°C overnight. The overnight culture was then diluted 100-fold into 2xYT and incubated at 37°C for another 2.5~3 h prior to the addition of IPTG (40 μM) to induce protein expression. After IPTG induction at 30°C for 4 h, cells (~30 μL) were directly mixed with 6× SDS loading buffer (6 μL) and subsequently analyzed by 10% SDS-PAGE and Western blot. The full length GST-TFR-PR-FLAG precursor and mature protease (PR-FALG) along with processing intermediates were detected with mouse anti-FLAG antibody (Sigma, St. Luis, MO).

## Competing interests

The authors declare that they have no competing interests.

## Authors' contributions

CC designed the project and wrote the manuscript. LH constructed the plasmids used in this study, performed 293T transfection and western blot analyses. AH carried out the *E. coli *protease maturation assay and participated in sequencing analysis of the constructs. All authors read and approved the final manuscript.
